# Stochastic Gradient Descent for Kernel-Based Maximum Correntropy Criterion

**DOI:** 10.3390/e26121104

**Published:** 2024-12-17

**Authors:** Tiankai Li, Baobin Wang, Chaoquan Peng, Hong Yin

**Affiliations:** 1 School of Mathematics and Statistics, South-Central MinZu University, Wuhan 430074, China; 2023110252@mail.scuec.edu.cn (T.L.); wbb1818@126.com (B.W.); 3085807@mail.scuec.edu.cn (C.P.); 2 School of Mathematics, Renmin University of China, Beijing 100872, China

**Keywords:** stochastic gradient descent, maximum correntropy criterion, non-Gaussian, convergence rate, 62J02

## Abstract

Maximum correntropy criterion (MCC) has been an important method in machine learning and signal processing communities since it was successfully applied in various non-Gaussian noise scenarios. In comparison with the classical least squares method (LS), which takes only the second-order moment of models into consideration and belongs to the convex optimization problem, MCC captures the high-order information of models that play crucial roles in robust learning, which is usually accompanied by solving the non-convexity optimization problems. As we know, the theoretical research on convex optimizations has made significant achievements, while theoretical understandings of non-convex optimization are still far from mature. Motivated by the popularity of the stochastic gradient descent (SGD) for solving nonconvex problems, this paper considers SGD applied to the kernel version of MCC, which has been shown to be robust to outliers and non-Gaussian data in nonlinear structure models. As the existing theoretical results for the SGD algorithm applied to the kernel MCC are not well established, we present the rigorous analysis for the convergence behaviors and provide explicit convergence rates under some standard conditions. Our work can fill the gap between optimization process and convergence during the iterations: the iterates need to converge to the global minimizer while the obtained estimator cannot ensure the global optimality in the learning process.

## 1. Introduction

Non-Gaussian noise and outliers are ubiquitous in many real-life applications, and robustness analysis plays an important role in the fields of signal processing and machine learning [1,2,3,4,5,6,7,8,9]. For regression problems, the classical least squares (LS) method is the most widely used tool. The LS method refers to the framework of second-order statistics due to its minimization of the variance of the prediction error, and the success of the LS method depends heavily on the assumption of Gaussianity. However, the LS method has poor performance when data may be contaminated by non-Gaussian noise or outliers. This motivates the application of MCC into robust regression problems as the correntropy loss can capture high-order moment information contained in the data. Due to its robustness to non-Gaussian noise, MCC has found wide applications in solving optimization problems in various scientific and engineering areas such as signal processing, regression analysis, feature selection, and data clustering; see [10,11,12,13,14,15,16,17,18,19,20,21] and the references therein.

Stochastic gradient descent (SGD) is a popular optimization method to solve large-scale computation problems due to its low memory requirement, computational complexity, and promising learning performances; see [22,23,24]. As an iterative method, SGD calculates a gradient randomly based on a random event and updates iterates along the direction of the negative gradient. Theoretical properties of SGD are well studied for optimization with both convex and strongly convex loss functions, but the existing theoretical results in the non-convex setting are not mature. Recently, non-convex optimization with SGD has attracted significant interest from researchers regarding the development of deep neural nets. In the paper, we employ SGD to solve the optimization problems induced by kernel-based MCC. As the correntropy loss is not convex, a rigorous theoretical foundation for applying SGD to MCC is not clear and will be investigated in the work.

Kernel methods are efficient tools for dealing with non-linear features in models. Here, the learning process of the kernel MCC method is associated with a Mercer kernel K. The function K:X×X→R is said to be a Mercer kernel if it is continuous, symmetric and positive semidefinite on X×X. It induces a reproducing kernel Hilbert space (RKHS) $H_{K}$ [25], which is defined to be the completion of the linear span of $\left\{K_{x}=K\left(\cdot ,x\right),x\in X\right\}$ with the inner product ${\langle K\left(\cdot ,x\right),K\left(\cdot ,u\right)\rangle }_{K}=K\left(x,u\right),\forall x,u\in X$. and the *reproducing property*

(1) $$\begin{array}{c} f\left(x\right)={\langle K\left(\cdot ,x\right),f\rangle }_{K},\;\forall f\in H_{K},\;x\in X. \end{array}$$

So, the following holds

(2) $$\begin{array}{c} {∥f∥}_{\infty }\leq \underset{x\in X}{\sup}\sqrt{K(x,x)}{∥f∥}_{K},\;\forall f\in H_{K}. \end{array}$$

In this paper, we attempt to present the convergence analysis of the kernel version of SGD applied to the correntropy loss,

$$\phi_{\sigma }\left(u\right)=\exp\left\{-\frac{u^{2}}{\sigma^{2}}\right\},\;u\in R,$$

where σ>0 is the robustness parameter. Assume $X\in R^{d}$ is a vector of explanatory variables, Y∈R is the response variable. Given a prediction function *f*, the correntropy loss $\phi_{\sigma }$ provides a measure of the error prediction error E=f(X)−Y, that is

(3) $$\begin{array}{c} \phi_{\sigma }\left(E\right)=\exp\left\{-\frac{{\left(f\left(X\right)-Y\right)}^{2}}{\sigma^{2}}\right\},\;\sigma >0. \end{array}$$

The purpose of the maximum correntropy criterion is to find a good predictor by maximizing (3). Given a set of independent and identically distributed (i.i.d.) observations, $z={\left\{\left(x_{i},y_{i}\right)\right\}}_{i=1}^{T},$ the implementation of MCC by SGD in an RKHS $H_{K}$ is defined with an initial value of $f_{1}=0$ and the updating rule

(4) $$\begin{array}{c} f_{t+1}=f_{t}-\eta_{t}\frac{2u_{t}}{\sigma^{2}}\exp\left\{-\frac{u_{t}^{2}}{\sigma^{2}}\right\}K_{x_{t}},\;t=1,\cdots ,T \end{array}$$

where $u_{t}=f\left(x_{t}\right)-y_{t}$ and $\eta_{t}>0$ are the step size.

In a previous work [7,9], an analysis of the implementation of MCC mainly focuses on the convergence of $\left\{f_{t}\right\}$ to the regression function E(Y|X=x), which is the value of the conditional mean of the output *Y*. However, in a non-parametric context, the target function of MCC varies with the choice of the robustness parameter σ. In the following paper [26,27], the authors established a sound theoretical foundation for MCC and provided a full analysis of the prediciton ability of MCC. They showed that MCC indeed belonged to the robust regression scheme when σ was large enough, but when σ→0, the target function of MCC was a modal regression fucntion; that is, $\max_{y\in Y}p(y|X=x)$ (p(·|X=x) denotes the condtional density function). Thus, this paper will investigate the convegence ability of SGD applied to MCC for any σ>0.

The contributions of the work are as follows:

(a) This work establishes the theoretical foundations for SGD applied to MCC. Some important convergence properties are provided, in which the role of the robustness parameter σ is presented.
(b) We introduce the Polyak–Łojasiewicz (PL) condition to derive the explicit convergence rates of algorithm (4). A global linear convergence rate is achieved when the step size $\eta_{t}$ is chosen properly.

The rest of the paper is organized as follows. In Section 2, after introducing necessary notations and assumptions that underlie our analysis, we provide and discuss our main theorems. Some discussions on the kernelized MCC are provided in Section 3. We provide the simulations in Section 4. Section 5 is devoted to the technical proof. We conclude this paper with Section 6.

## 2. Main Results

This paper investigates the convergence of algorithm (4) in a non-parametric context. Some necessary notations and assumptions are given. Let ρ be a Borel probability measure on the product space Z:=X×Y. The samples $z={\left\{z_{i}:=\left(x_{i},y_{i}\right)\right\}}_{i=1}^{T}\subset Z$ are drawn independently according to ρ. Let $\rho_{X}$ be the marginal distribution of ρ on X, and ρ(y|x) be the conditional distribution on Y given x∈X. Associated with $\left(\phi_{\sigma },\rho \right)$, the *generalization error*E for f:X→Y is given by

$$\begin{array}{c} E\left(f\right)=\int_{Z}\phi_{\sigma }\left(f\left(x\right)-y\right)d\rho , \end{array}$$

which is well defined for any measurable function *f*. We assume that there exists a function $f^{*}$ that maximizes the expected correntropy error. In this sense, the goal of MCC learning is to find an approximation of $f^{*}$ through only the training set $z={\left\{z_{i}:=\left(x_{i},y_{i}\right)\right\}}_{i=1}^{T}$ when measure ρ is unknown. The prediction ability to algorithm (4) is estimated by $E\left(f^{*}\right)-E\left(f_{T+1}\right)$. In the rest of the work, we denote the gradient of the functional E(f) in a RKHS $H_{K}$ by ∇E(f).

**Theorem 1.**  *Define $\left\{f_{t}\right\}$ by algorithm (4) and take the step size $\sum_{t=1}^{\infty }\eta_{t}^{2}<\infty$. Then, the following statements holds.*

*(a)*  *The generalization error $E\left(f^{*}\right)-EE\left(f_{t+1}\right)$ is uniformly bounded. More precisely, there is some constant C, such that*
$$E\left(f^{*}\right)-EE\left(f_{t+1}\right)\leq C\left(1+\sigma^{-4}\right),\;t=1,\cdots ,T.$$
*(b)*  *There is some constant C>0, such that*
$$\underset{t=1,\cdots ,T}{\inf}E{∥\nabla E\left(f_{t}\right)∥}_{K}^{2}\leq C\left(1+\sigma^{-4}\right){\left(\sum_{t=1}^{T}\eta_{t}\right)}^{-1},$$

*where the constant C will be given explicitly in the proof.*

**Remark 1.**  *Statement (a) tells us that the error $E\left(f^{*}\right)-EE\left(f_{t}\right)$ converges to a bounded random variable without any boundness requirement on $f_{t}$ appearing in previous work [22,28]. That is because of the so-called redescending property of the correntropy loss. We say that loss ℓ(·) satisfies the redescending property if $\ell^{\prime }\left(\cdot \right)$ is non-decreasing near the origin, but decreasing towards 0 far from the origin. At present, there are a great deal of papers to describe the redescending property in robustness analysis [29,30].*

*Statement (b) shows that after T iterations, there exists a single iterate, such that its gradient converges to 0 if we choose the step size $\sum_{t=1}^{\infty }\eta_{t}=\infty .$ Explicit rates are provided in the following corollary by instantiating the step sizes.*

**Corollary 1.**  *Define $\left\{f_{t}\right\}$ by algorithm (4), we have*

*(a)*  *If $\eta_{t}=t^{-1+\epsilon }$ for any 0<ϵ<1, then*
$$\begin{array}{c} \underset{t=1,\cdots ,T}{\inf}E{∥\nabla E\left(f_{t}\right)∥}_{K}^{2}=O\left(T^{-1+\epsilon }\right). \end{array}$$
*(b)*  *If $\eta_{t}=t^{-\frac{1}{2}}\log t$, then*
$$\begin{array}{c} \underset{t=1,\cdots ,T}{\inf}E{∥\nabla E\left(f_{t}\right)∥}_{K}^{2}=O\left(T^{-\frac{1}{2}}\log T\right). \end{array}$$

As we see, the convergence rate of algorithm (4) varies with the step size and can achieve the order $O(T^{-1})$, as presented in statement (a).

In the following, we improve our convergence analysis by imposing the Polyak–Łojasiewicz (PL) condition, which has been explored to show fast convergence rates for SGD without convexity or strong convexity, see [31,32].

**Assumption 1.**  *We say that E(f) satisfies Polyak–Łojasiewicz (PL) condition if the following holds for some μ>0*

(5) $$\begin{array}{c} E\left(f^{*}\right)-E\left(f\right)\leq \mu^{-1}{∥\nabla E\left(f\right)∥}_{\infty }^{2} \end{array}$$

*for any measurable function f:X→Y.*

This condition was originally introduced by Polyak [33] and is commonly used in non-convex gradient descent analysis [32]. It simply requires that the gradient grows faster than a quadratic distance between the generalization error, and is a sufficient condition to guarantee a global linear convergence rate for the gradient descent. In this work, we use the PL condition to provide a full analysis of SGD for MCC implementation.

**Theorem 2.**  *Define $\left\{f_{t}\right\}$ by algorithm (4) and suppose (5) holds.*

*(a)*  *If $\sum_{t=1}^{\infty }\eta_{t}^{2}<\infty$ and $\sum_{t=1}^{\infty }\eta_{t}=\infty$, then*
$$\lim_{t\to \infty }E\left(f^{*}\right)-EE\left(f_{t+1}\right)=0.$$
*(b)*  *If $\sum_{t=1}^{\infty }\eta_{t}^{2}<\infty$ and $\sum_{t=1}^{\infty }\eta_{t}=\infty$, then*
$$\lim_{t\to \infty }{∥\nabla E\left(f_{t}\right)∥}_{K}^{2}=0.$$
*(c)*  *If there exists some $t_{0}\in N$, such that for any $t\geq t_{0}$, $\eta_{t}\leq \frac{\sigma^{2}}{4}$, then*
$$\begin{array}{c} E\left(f^{*}\right)-EE\left(f_{t+1}\right)\leq \prod_{j=1}^{t}\left(1-\frac{\eta_{j}\kappa^{-2}\mu }{2}\right)\left(E\left(f^{*}\right)-E\left(f_{1}\right)\right). \end{array}$$

**Remark 2.**  *Statements (a) and (b) establish sufficient conditions to guarantee almost certain convergence in SGD applied to MCC. Statement (c) tells us that with a constant step size of $\eta_{t}\equiv \frac{\sigma^{2}}{4}$, algorithm (4) has a global linear convergence rate $O\left({\left(1-\frac{\sigma^{2}\kappa^{-2}\mu }{8}\right)}^{T}\right)$. It also shows that the value of the robustness parameter σ plays an important role in the choice of stepsize for deriving fast convergence rates.*

## 3. Discussions

Recent theoretical works on the global solutions to SDG generated by nonconvex losses are limited. Inspired by the work of [34,35], MCC is based on a smooth loss that can be utilized in the theoretical analysis and sufficient convergence conditions can be derived for MCC. Thus, a fine theoretical foundation kernel-based MCC is established in the work, allthough no convexity property assumption has been imposed on the optimization problems. We show that the usual bounded gradient assumptions on nonconvex learning can be removed without affecting the convergence rates. By using a condition referred to as PL inequality, we find that the last iteration of SGD with kernel-based MCC can enjoy fast convergence speeds. To the best of our knowledge, this work provides the first theoretical analysis of MCC that establishes a global linear convergence rate.

We review some work related to the kernelized MCC. In linear additive noise models, the studies by [7,26,35] investigated regression models associated with correntropy-induced losses, demonstrating that the scale parameter in the learning process balances the convergence rates of the regression model with its robustness. However, their approaches rely solely on batch algorithms, which are limited in streaming data scenarios and large-scale computational settings. Moreover, the convergence rates achieved in their work are noticeably inferior to those established in our study (see Theorem 2).

We turn to the theoretical works on the gradient descent in training kernelized MCC. In the works of [10,21,36], the authors focussed on the learning performance and convergence ability in terms of the target functions, defined as the conditional mean E(Y|X=x). But, it has been proved in [26] that in MCC models, for a good generalization ability and robustness properties, the minimizer of the *generalization error* E(f) is not necessarily E(Y|X=x). Our work does not impose extra conditions on the target functions and can apply to general learning schemes. In addition, our analysis relaxes thethe requirement for the choice of σ, which must be sufficiently large in [10,21,36].

## 4. Simulation Validation

In this section, we provide some experimental results to confirm our theoretical results. These experiments are simulated from the regression model $y_{i}=f^{*}\left(x_{i}\right)+\epsilon$, where $f^{*}\left(x_{i}\right)=x_{i}\left(1-x_{i}\right)$ and the random samples $x_{i}$ are drawn independently according to the uniform distribution U[−1,2]. To carry out the experiments, the noise ϵ is generated as follows.

1. Gaussion noise: ϵ∼$N(0,0.1^{2});$
2. outlier noise: 90% noise ∼$N(0,0.1^{2})$, 10% noise ∼U[−1,1];
3. skewed noise: $\epsilon =z_{1}-z_{2}$, where $z_{1},z_{2}∼Exp\left(0.2\right);$
4. GMM noise: 90% noise ∼$N(0,0.1^{2})$, 10% noise is generated by the following density probability

$$\begin{array}{c} p\left(\epsilon \right)=\frac{1}{2\sqrt{2\pi }\times 0.1}\exp\left\{-\frac{{\left(\epsilon -0.5\right)}^{2}}{2\times 0.1^{2}}\right\}+\frac{1}{2\sqrt{2\pi }\times 0.1}\exp\left\{-\frac{{\left(\epsilon +0.5\right)}^{2}}{2\times 0.1^{2}}\right\}. \end{array}$$

**Figure 1:**
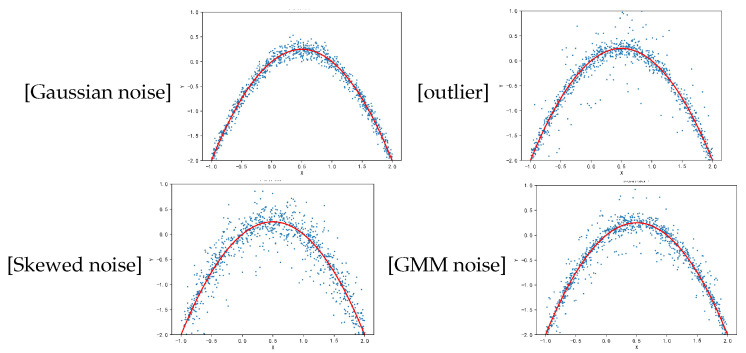
Comparison of four types of noises with 1000 samples.

We generate a dataset of 1000 samples for the above four types of noise. See Figure 1.

**Figure 2:**
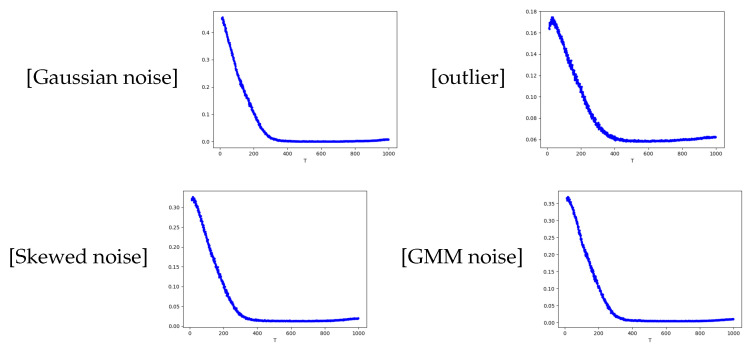
Comparison of generalization errors for different noises. The vertical coordinate denotes generalization errors and the horizontal coordinate denotes the iteration time.

We chose the Gaussian kernel function $G\left(x,u\right)=\exp\left\{-\frac{{∥x-u∥}^{2}}{2}\right\}$ for the kernel MCC. We applied SGD to the kernel MCC with stepsizes of η=0.1 and σ=0.1. For each type of noise, we ran the SGD for *T* = 1000 times. The generalization errors $E(f_{t})$ with iterations *t* are plotted in Figure 2. It is observed that for different types of noise, the generalization errors decreased rapidly as iterations progressed. This shows the efficiency of SGD for MCC in dealing with non-Gaussain noise. This coincides with our theory.

## 5. Proofs

Now, we provide the proofs for our main results.

**Proof of Theorem 1.**  As $\phi_{\sigma }^{\prime }\left(u\right)=-\frac{2u}{\sigma^{2}}\exp\left\{-\frac{u^{2}}{\sigma^{2}}\right\}$, we know

(6) $$\begin{array}{c} |\phi_{\sigma }^{\prime }\left(u\right)-\phi_{\sigma }^{\prime }\left(v\right)|\leq \frac{4}{\sigma^{2}}\left|u-v\right|,\;u,v\in R. \end{array}$$

It follows that for any u,v∈R,

(7) $$\begin{array}{c} -\phi_{\sigma }\left(u\right)\leq -\phi_{\sigma }\left(v\right)+\phi_{\sigma }^{\prime }\left(v\right)\left(u-v\right)+\frac{2}{\sigma^{2}}{\left|u-v\right|}^{2}. \end{array}$$

Applying the inequality above with $u=f_{t+1}\left(x\right)-y$, $v=f_{t}\left(x\right)-y$, then by (1)

(8) $$\begin{array}{cc} -\phi_{\sigma }\left(f_{t+1}\left(x\right)-y\right) & \leq -\phi_{\sigma }\left(f_{t}\left(x\right)-y\right)+\phi_{\sigma }^{\prime }\left(f_{t}\left(x\right)-y\right)\left(f_{t+1}\left(x\right)-f_{t}\left(x\right)\right)+\frac{2}{\sigma^{2}}{\left|f_{t+1}\left(x\right)-f_{t}\left(x\right)\right|}^{2}\\ \; & \leq \phi_{\sigma }\left(f_{t}\left(x\right)-y\right)+{\langle f_{t+1}-f_{t},\phi_{\sigma }^{\prime }\left(f_{t}\left(x\right)-y\right)K_{x}\rangle }_{K}+\frac{2}{\sigma^{2}}{\left|f_{t+1}\left(x\right)-f_{t}\left(x\right)\right|}^{2}. \end{array}$$

Taking the expectation with respect to z=(x,y) on both sides, and using (4)

(9) $$\begin{array}{cc} -E(f_{t+1}) & \leq -E\left(f_{t}\right)-{\langle f_{t+1}-f_{t},-\nabla E\left(f_{t}\right)\rangle }_{K}+\frac{2\eta_{t}^{2}}{\sigma^{2}}\int_{Z}{\left|\frac{2u_{t}}{\sigma^{2}}\exp\left\{-\frac{u_{t}^{2}}{\sigma^{2}}\right\}K_{x_{t}}\right|}^{2}d\rho \\ \; & =-E\left(f_{t}\right)-{\langle -\eta_{t}\phi_{\sigma }^{\prime }\left(f\left(x_{t}\right)-y_{t}\right)K_{x_{t}},-\nabla E\left(f_{t}\right)\rangle }_{K}+\frac{2\eta_{t}^{2}}{\sigma^{2}}\int_{Z}|\phi_{\sigma }^{\prime }\left(f_{(}x_{t}\right)-y_{t}{)K_{x_{t}}|}^{2}d\rho . \end{array}$$

For the term $|\phi_{\sigma }^{\prime }\left(f_{(}x_{t}\right)-y_{t}{)K_{x_{t}}|}^{2}$, using (2), we know

(10) $$\begin{array}{c} |\phi_{\sigma }^{\prime }\left(f_{(}x_{t}\right)-y_{t})K_{x_{t}}{|}^{2}\leq \kappa^{2}|\phi_{\sigma }^{\prime }\left(f_{(}x_{t}\right)-y_{t}{)|}^{2}=\frac{4u_{t}^{2}\kappa^{2}}{\sigma^{4}}\exp\left\{-\frac{2u_{t}^{2}}{\sigma^{2}}\right\}\leq \frac{2\kappa^{2}}{\sigma^{2}}. \end{array}$$

where the last inequality is obtained by $te^{-t}\leq 1$ for any t>0. Putting it into (9) yields

$$\begin{array}{c} -E\left(f_{t+1}\right)\leq -E\left(f_{t}\right)-{\langle -\eta_{t}\phi_{\sigma }^{\prime }\left(f\left(x_{t}\right)-y_{t}\right)K_{x_{t}},-\nabla E\left(f_{t}\right)\rangle }_{K}+\frac{4\kappa^{2}\eta_{t}^{2}}{\sigma^{4}}. \end{array}$$

Noting that $f_{t}$ is independent of $z_{t}$, then by taking the conditional expectation with respect to $z_{t}$, we derive

$$\begin{array}{c} -EE\left(f_{t+1}\right)\leq -E\left(f_{t}\right)-\eta_{t}{∥\nabla E\left(f_{t}\right)∥}_{K}^{2}+\frac{4\kappa^{2}\eta_{t}^{2}}{\sigma^{4}} \end{array}$$

Taking the conditional expectation with respect to $z_{t-1},z_{t-1},\cdots ,z_{1}$ sequentially yields

(11) $$\begin{array}{c} -EE\left(f_{t+1}\right)\leq -EE\left(f_{t}\right)-\eta_{t}E{∥\nabla E\left(f_{t}\right)∥}_{K}^{2}+\frac{4\kappa^{2}\eta_{t}^{2}}{\sigma^{4}}. \end{array}$$

This implies that

$$\begin{array}{c} E\left(f^{*}\right)-EE\left(f_{t+1}\right)\leq E\left(f^{*}\right)-EE\left(f_{t}\right)+\frac{4\kappa^{2}\eta_{t}^{2}}{\sigma^{4}}. \end{array}$$

An application of the inequality recursively provides

$$E\left(f^{*}\right)-EE\left(f_{t+1}\right)\leq E\left(f^{*}\right)-EE\left(f_{1}\right)+\frac{4\kappa^{2}}{\sigma^{4}}\sum_{j=1}^{t}\eta_{j}^{2}.$$

So, we can obtain that for any t∈N,

(12) $$\begin{array}{cc} E\left(f^{*}\right)-EE\left(f_{t+1}\right) & \leq E\left(f^{*}\right)-EE\left(f_{1}\right)+\frac{4\kappa^{2}}{\sigma^{4}}\sum_{j=1}^{t}\eta_{j}^{2}\\ \; & =C(1+\sigma^{-4})<\infty \end{array}$$

where $C:=\max\{1,4\kappa^{2}\sum_{j=1}^{\infty }\eta_{j}^{2}\}$ and the last inequality is obtained by E(f)≤1 for any measurable function f:X→Y.

Then, statement (a) holds.

Now, we provide statement (b). Inequality (11) also provides

$$\begin{array}{c} \eta_{t}E{∥\nabla E\left(f_{t}\right)∥}_{K}^{2}\leq EE\left(f_{t+1}\right)-EE\left(f_{t}\right)+\frac{4\kappa^{2}}{\sigma^{4}}\eta_{t}^{2},\;t=1,\cdots ,T. \end{array}$$

A summation of the aforementioned inequality then implies

$$\begin{array}{cc} \sum_{j=1}^{t}\eta_{t}E{∥\nabla E\left(f_{j}\right)∥}_{K}^{2} & \leq \sum_{j=1}^{t}EE\left(f_{j+1}\right)-EE\left(f_{j}\right)+\frac{4\kappa^{2}}{\sigma^{4}}\sum_{j=1}^{t}\eta_{j}^{2}\leq EE\left(f_{t+1}\right)+\frac{4\kappa^{2}}{\sigma^{4}}\sum_{j=1}^{t}\eta_{j}^{2}\\ \; & \leq 1+\frac{4\kappa^{2}}{\sigma^{4}}\sum_{j=1}^{t}\eta_{j}^{2}:=C\left(1+\sigma^{-4}\right)<\infty . \end{array}$$

So, we obtain

$$\underset{j=1,\cdots ,t}{\inf}E{∥\nabla E\left(f_{j}\right)∥}_{K}^{2}\sum_{j=1}^{t}\eta_{j}\leq C\left(1+\sigma^{-4}\right).$$

Then, statement (b) holds. □

**Proof of Corollary 1.**  We know that for any t∈N,

$$\begin{array}{c} \frac{1}{1-\gamma }\left[{\left(t+1\right)}^{1-\gamma }-1\right]\leq \sum_{j=1}^{t}j^{-\gamma }\leq \frac{1}{1-\gamma }t^{1-\gamma },\;\gamma \in \left(0,1\right). \end{array}$$

Using statement (b) of Theorem 1 with γ=1−ϵ implies that statement (a) holds.

Statement (b) can be similarly proven. □

**Proof of Theorem 2.**  For the proof of statement (a), we use estimate (11). Adding $E(f^{*})$ on both sides provides

$$\begin{array}{c} E\left(f^{*}\right)-EE\left(f_{t+1}\right)\leq E\left(f^{*}\right)-EE\left(f_{t}\right)-\eta_{t}E{∥\nabla E\left(f_{t}\right)∥}_{K}^{2}+\frac{4\kappa^{2}\eta_{t}^{2}}{\sigma^{4}}. \end{array}$$

Note that the relation (2), then $∥\nabla E\left(f_{t}\right){∥}_{\infty }\leq \kappa {∥\nabla E\left(f_{t}\right)∥}_{K}$ This together with (5) yields

$$\begin{array}{cc} E\left(f^{*}\right)-EE\left(f_{t+1}\right) & \leq E\left(f^{*}\right)-EE\left(f_{t}\right)-\kappa^{-2}\eta_{t}E{∥\nabla E\left(f_{t}\right)∥}_{\infty }^{2}+\frac{4\kappa^{2}\eta_{t}^{2}}{\sigma^{4}}\\ \; & \leq E\left(f^{*}\right)-EE\left(f_{t}\right)-\kappa^{-2}\mu \eta_{t}\left(E\left(f^{*}\right)-EE\left(f_{t}\right)\right)+\frac{4\kappa^{2}\eta_{t}^{2}}{\sigma^{4}}\\ \; & \leq \left(1-\mu^{-2}\eta_{t}\right)\left(E\left(f^{*}\right)-EE\left(f_{t}\right)\right)+\frac{4\kappa^{2}\eta_{t}^{2}}{\sigma^{4}}. \end{array}$$

Using the inequality recursively from t=1 to *T*, then

$$\begin{array}{cc} E\left(f^{*}\right)-EE\left(f_{T+1}\right) & \leq \sum_{t=1}^{T}\left(1-\kappa^{-2}\eta_{t}\right)\left(E\left(f^{*}\right)-EE\left(f_{1}\right)\right)+\frac{4\kappa^{2}}{\sigma^{4}}\sum_{t=1}^{T}\eta_{t}^{2}\prod_{j=t+1}^{T}\left(1-\kappa^{-2}\eta_{j}\right)\\ \; & \leq \exp\left\{-\kappa^{-2}\sum_{t=1}^{T}\eta_{t}\right\}\left(E\left(f^{*}\right)-EE\left(f_{1}\right)\right)+\frac{4\kappa^{2}}{\sigma^{4}}\sum_{t=1}^{T}\eta_{t}^{2}\prod_{j=t+1}^{T}\left(1-\kappa^{-2}\eta_{j}\right) \end{array}$$

The part of the first term $\lim_{T\to \infty }\exp\left\{-\kappa^{-2}\sum_{t=1}^{T}\eta_{t}\right\}=0$ due to $\sum_{t=1}^{\infty }\eta_{t}=\infty$. The part of the second term $\sum_{t=1}^{T}\eta_{t}^{2}\prod_{j=t+1}^{T}$ goes to zero, as T→∞ due to Lemma 11 in [37].

The the proof of (a) is complete.

For the proof of statement (b), we recall an elementary inequality in [24]. For any positive functional $\ell \left(f\right),f\in H_{K}$, if the gradient of *ℓ* is *L*-Lipschitz continuous with respect to $H_{K}$, then

(13) $$\begin{array}{c} {∥\Delta \ell \left(u\right)∥}_{K}^{2}\leq 2L\ell \left(u\right). \end{array}$$

Let $\ell \left(f\right)=E\left(f^{*}\right)-E\left(f\right)$, then by (6), we obtain

$$\begin{array}{c} \ell^{\prime }\left(f\right)=\int_{Z}-\phi_{\sigma }^{\prime }\left(f\left(x\right)-y\right)K_{x}d\rho \end{array}$$

and for any $f,g\in H_{K}$, it follows that

$$|\ell^{\prime }\left(f\right)-\ell^{\prime }\left(g\right)|\leq \int_{z}\frac{4}{\sigma^{2}}\left|f\left(x\right)-g\left(x\right)\right|K_{x}d\rho \leq \frac{4\kappa^{2}}{\sigma^{2}}{∥f-g∥}_{K}$$

by using (2). The gradient of the functional ℓ(f) is $\frac{4\kappa^{2}}{\sigma^{2}}$-Lipschitz continuous in $H_{K}.$

Note that $\nabla E\left(f\right)=\nabla \left(E\left(f\right)-E\left(f^{*}\right)\right)=\int_{z}-\phi_{\sigma }^{\prime }\left(f\left(x\right)-y\right)K_{x}d\rho$ as $\nabla E(f^{*})=0$. Then, we obtain

(14) $$\begin{array}{c} ∥\nabla \left(E\left(f\right)-E\left(f^{*}\right)\right){∥}_{K}^{2}={∥\nabla E\left(f\right)∥}_{K}^{2}\leq \frac{8\kappa^{2}}{\sigma^{2}}\left(E\left(f^{*}\right)-E\left(f\right)\right) \end{array}$$

using (13) with $L=\frac{4\kappa^{2}}{\sigma^{2}}$. Replacing *f* with $f_{t}$, then from statement (a), we can derive

$$\begin{array}{c} \lim_{t\to \infty }{∥\nabla E\left(f_{t}\right)∥}_{K}^{2}\leq \frac{8\kappa^{2}}{\sigma^{2}}\lim_{t\to \infty }\left(E\left(f^{*}\right)-E\left(f_{t}\right)\right)=0. \end{array}$$

Then, the proof of statement (b) is complete.

For the proof of statement (c), we take the expectation with respect to $z_{1},\cdots ,z_{t}$ and add $E(f^{*})$ on both sides of (9). So, we have

(15) $$\begin{array}{cc} E\left(f^{*}\right)-EE\left(f_{t+1}\right) & \leq E\left(f^{*}\right)-EE\left(f_{t}\right)-\eta_{t}E∥\nabla E\left(f_{t}\right){∥}_{K}^{2}+\frac{2\eta_{t}^{2}}{\sigma^{2}}E{∥\nabla E\left(f_{t}\right)∥}_{K}^{2}. \end{array}$$

When there exists $t_{0}$, such that for any $t\geq t_{0}$, $\eta_{t}\leq \frac{\sigma^{2}}{4}$, we have $\frac{2\eta_{t}^{2}}{\sigma^{2}}\leq \frac{1}{2}\eta_{t}$ and

(16) $$\begin{array}{c} E\left(f^{*}\right)-EE\left(f_{t+1}\right)\leq E\left(f^{*}\right)-EE\left(f_{t}\right)-\frac{\eta_{t}}{2}E{∥\nabla E\left(f_{t}\right)∥}_{K}^{2},\;t>t_{0}. \end{array}$$

Using (2) and (5) again, we obtain

$$E\left(f^{*}\right)-EE\left(f_{t+1}\right)\leq E\left(f^{*}\right)-EE\left(f_{t}\right)-\frac{\eta_{t}\kappa^{-2}}{2}E{∥\nabla E\left(f_{t}\right)∥}_{K}^{2}\leq \left(1-\frac{\eta_{t}\kappa^{-2}\mu }{2}\right)\left(E\left(f^{*}\right)-EE\left(f_{t}\right)\right).$$

Applying the estimate iteratively from t=1 to *T*, we obtain the conclusion for statement (c).

The proof is finished. □

## 6. Conclusions and Future Works

This paper studies the stochastic gradient method with correntropy loss functions. More precisely, we study how convergence properties can be achieved through a suitable choice of step size in robustness learning. We also use the PL condition to provide a global linear convergence rate. These results refine the previous work and provide a theoretical foundation for SGD with MCC.

Two related questions are worthwhile for future research. First, our analysis provides very useful insights on the application of SGD for MCC. The simulation is also consistent with our theory. However, the optimal selection of σ is unknown in practice. It is necessary to develop empirically applicable parameter selection strategies for optimal σ. Secondly, with the development of deep learning, various stochastic gradient methods are continuously emerging that accelerate the training progress, such as Adam [38], AdaGrad [39], RMSProp [40], and AMSGrad [41]. Non-convexity [42,43,44] of the MCC loss function will bring major challenges in the theoretical analysis. The techniques used in this paper may also be applied to other stochastic gradient methods. Meanwhile, our error bounds already imply linear convergence rates. It is yet unknown whether the non-convexity can be overcome in the error analysis and similar convergence results can be derived in other stochastic gradient methods. This will be considered in future research.

## Data Availability

The data supported by this study can be found in the article.
